# Anti-Inflammatory Properties of Plasma from Children with Short Bowel Syndrome

**DOI:** 10.3390/pathogens10081021

**Published:** 2021-08-13

**Authors:** Irshad Ahmed Hajam, Farhana Ali, Jocelyn Young, Mary Abigail Garcia, Christopher Cannavino, Nanda Ramchandar, George Y. Liu

**Affiliations:** 1 Department of Pediatrics, Division of Infectious Diseases, University of California, San Diego, CA 92123, USA; ihajam@health.ucsd.edu (I.A.H.); ccannavino@health.ucsd.edu (C.C.); 2 Department of Pediatrics, Division of Gastroenterology, University of California, San Diego, CA 92123, USA; faali@health.ucsd.edu (F.A.); jyoung@health.ucsd.edu (J.Y.); mag003@health.ucsd.edu (M.A.G.); 3 Rady Children’s Hospital, San Diego, CA 92123, USA

**Keywords:** *Escherichia coli*, plasma therapy, sepsis, short bowel syndrome, survival

## Abstract

Sepsis, resulting from a dysregulated host immune response to invading pathogens, is the leading cause of mortality in critically ill patients worldwide. Immunomodulatory treatment for sepsis is currently lacking. Children with short bowel syndrome (SBS) may present with less severe symptoms during gram-negative bacteremia. We, therefore, tested the hypothesis that plasma from children with SBS could confer protection against *Escherichia coli* sepsis. We showed that SBS plasma at 5% and 10% concentrations significantly (*p* < 0.05) inhibited the production of both TNF-α and IL-6 induced by either *E. coli-* or LPS-stimulated host cells when compared to plasma from healthy controls. Furthermore, mice treated intravenously with select plasma samples from SBS or healthy subjects had reduced proinflammatory cytokine levels in plasma and a significant survival advantage after *E. coli* infection. However, SBS plasma was not more protective than the plasma of healthy subjects, suggesting that children with SBS have other immunomodulatory mechanisms, in addition to neutralizing antibodies, to alleviate their symptoms during gram-negative sepsis.

## 1. Introduction

Short bowel syndrome (SBS) is a malabsorptive state that classically results from surgical resection of the small intestine for various reasons, including congenital anomalies of the gastrointestinal tract or acquired traumatic injuries such as necrotizing enterocolitis in infants [1,2,3]. Owing to the considerable intestinal loss and resultant inadequate absorption of fluids, electrolytes, and nutrients, parenteral nutrition (PN) is the gold standard for treatment to support growth and nutrition and is an essential life-saving intervention for most patients severely affected by SBS [4,5,6,7]. Although PN has dramatically improved the survival of SBS patients [7,8], its use negatively impacts gut barrier functions and mucosal immunity. PN-dependent SBS patients have been reported to have increased intestinal permeability, short bowel bacterial overgrowth (SBBO) and enteritis, reduced microbiome diversity, catheter-related bacterial sepsis, and gastrointestinal mucosal atrophy [7,9,10,11,12,13].

A critical function of the gut mucosal epithelium is to prevent bacterial translocation, a phenomenon in which gut resident pathogenic microbes and their components traverse to the underlying tissues via transcellular or paracellular pathways [14,15]. Animal models [16] and limited human studies [17,18,19,20] support the role of PN as an inducer of local or systemic inflammation concomitant with the gut barrier dysfunction and bacterial translocation. A study by Ziegler et al. showed that patients with SBS are serially and intermittently exposed to increased systemic levels of bacterial products, including flagellin and lipopolysaccharide (LPS), and serum antibody titers against these bacterial products are readily detectable [21]. LPS, an endotoxin, is recognized as the most potent microbial mediator involved in the pathogenesis of sepsis [22], which is a dysregulated host immune response to infections, leading to tissue damage and multiple organ failure [23]. In mice, the systemic inoculation of bacteria [24,25] or LPS [26] potently activates a lethal array of inflammatory mediators and results in a sepsis-like syndrome or death. Our institutional experience, in accordance with other reports describing children with SBS and other cohorts of bacteremic children, has been that children with SBS have high rates of bloodstream infections with sepsis-causing gram-negative (GN) bacterial species [27,28,29], yet some have clinically shown only modest symptoms with GN infections. Furthermore, both LPS and flagellin, which cause sepsis in otherwise healthy populations, have been detected in patients with SBS [30]. The phenomenon of tolerance to a sepsis-like situation in SBS patients could be attributable to either the presence of microbe-specific antibodies or impairments to T cell responses or, potentially, a combination of both. Limited human studies have shown that SBS patients have impaired T cell responses and poorly respond to antigenic stimulation [31], while competent T cells can increase sepsis morbidity and tissue injury [32]. Considering that SBS subjects are frequently exposed to systemic GN bacteria, we hypothesized that these children may have a robust anti-GN bacterial humoral response to shield them from the sepsis syndrome. Accordingly, we sought to determine whether plasma from SBS subjects would prevent *Escherichia coli* sepsis in a murine model. Such studies would explain the relative SBS subjects’ relative tolerance to GN sepsis and could lead to the development of novel interventions against GN sepsis and septic shock.

We used a well-characterized murine model of *E. coli* sepsis to test the protective effect of plasma from children with SBS against sepsis-induced cytokine inflammation and mortality. Our data demonstrate that SBS, as well as healthy plasma therapy in mice challenged with a lethal dose of *E. coli,* improved host survival. In vivo protection is consistent with the finding of plasma inhibition of cellular immune responses induced by LPS and multiple *E. coli* strains in vitro.

## 2. Results

### 2.1. Anti-Inflammatory Effect of Plasma from SBS and Healthy Subjects In Vitro

**Figure 1:**
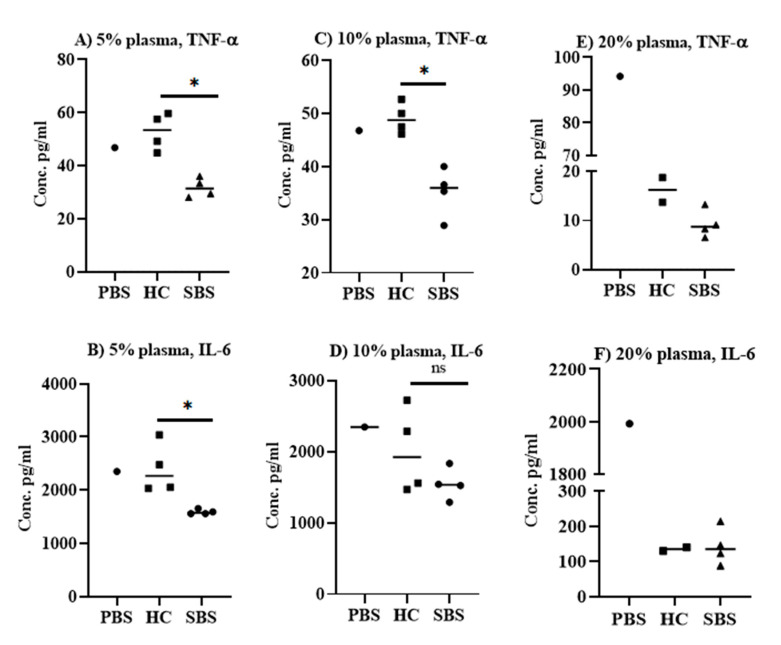
LPS neutralization in vitro. *E. coli* LPS was incubated with mouse splenocytes plus 5% (**A**,**B**), 10% (**C**,**D**) or 20% (**E**,**F**) concentration of plasma from SBS or HC subjects. TNF-α and IL-6 from culture supernatants were quantified by a solid-phase sandwich ELISA performed in duplicate or triplicate wells. (**A**,**B**): Pro-inflammatory cytokine inhibition by 5% plasma. (**C**,**D**): Pro-inflammatory cytokine inhibition by 10% plasma. (**E**,**F**): Pro-inflammatory cytokine inhibition by 20% plasma. Data are presented as median, and each datapoint represents one subject. A non-parametric Mann–Whitney T-test was applied to analyze the significant difference. * *p* < 0.05. SBS: short bowel syndrome, HC: healthy controls.

**Figure 2:**
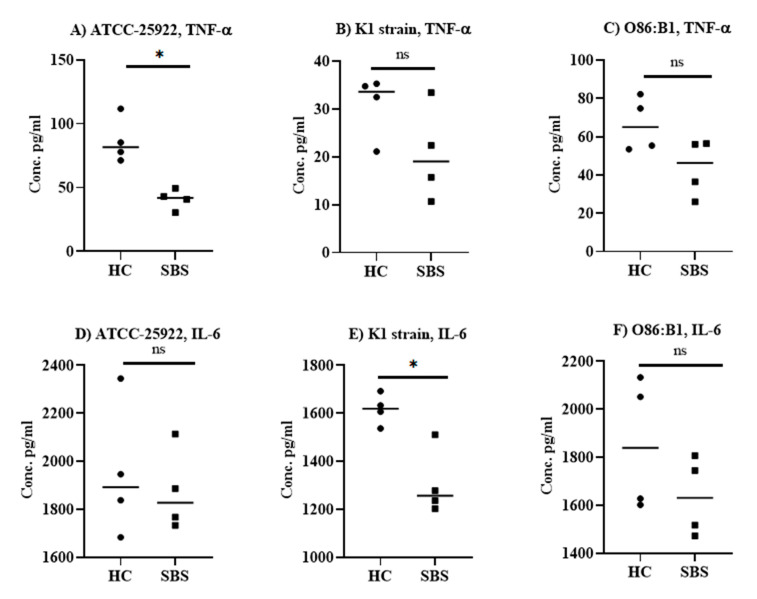
Anti-inflammatory effect of plasma from SBS or HC subjects in *E. coli*-stimulated splenocyte cultures. *E. coli* strains ATCC 25922 (**A**,**D**), K1 RS218 (**B**,**E**), and O86:B1 (**C**,**F**) were incubated with mouse splenocytes plus 20% plasma from either SBS or HC subjects. TNF-α and IL-6 in culture supernatants were quantified by ELISA, performed in either duplicate or triplicate wells. Data are presented as median, and each datapoint represents one subject. A non-parametric Mann–Whitney T-test was applied to analyze the statistical significance. * *p* < 0.05. SBS; short bowel syndrome, HC; healthy controls.

The effect of plasma from SBS and healthy subjects on LPS- and *E. coli*-mediated cellular responses was evaluated in splenocyte stimulation assays. Notably, the SBS plasma samples were derived from children, and plasma from healthy controls (HC) were from adults. Splenocytes from CD1 mice were incubated with plasma from SBS or HC, along with either LPS or *E. coli*. The production of TNF-α and IL-6 in culture supernatant was measured by ELISA. The results showed that the inhibition of TNF-α and IL-6 production varied depending on the cytokine being examined, the concentration of plasma sample used to neutralize the LPS (Figure 1), and the *E. coli* strain used for the stimulation of cells (Figure 2). All plasma samples from subjects with SBS at both 5% (Figure 1A,B) and 10% concentrations (Figure 1C,D) significantly (*p* < 0.05) inhibited the ability of LPS to stimulate TNF-α (~1.4–1.6 fold) and IL-6 (1.3–1.5 fold) secretion compared to the HC, which exhibited comparable cytokine levels to cells stimulated by LPS alone (Supplementary Figure S1A–D). At a 20% concentration, however, plasma from either SBS or HC markedly suppressed TNF-α and IL-6 secretion by splenocytes stimulated with LPS. Compared to cells stimulated with LPS alone, TNF-α levels were 14.5- and 8.3-fold lower with the plasma samples from SBS and the HC subjects, respectively (Figure 1E). The IL-6 levels were equally inhibited by SBS and HC samples, and were ~14-fold lower than the cells stimulated with LPS alone (Figure 1F). As a baseline control, we measured IL-6 and TNF-α levels in plasma samples by ELISA. Both IL-6 and TNF-α were undetectable in SBS and HC plasma samples (data not shown). Overall, our results indicate that the plasma from either SBS or healthy subjects has the ability to neutralize the proinflammatory activities of LPS, though plasma from SBS subjects is relatively more effective than plasma from HC subjects.

Next, we tested the effect of plasma from SBS and HC subjects on the inflammation induced by *E. coli* co-incubated with mouse splenocytes in vitro. We showed that both SBS and HC plasma samples significantly (*p* < 0.05) inhibited TNF-α production induced by the respective *E. coli* strains (Figure S2A–C), although a more profound inhibition of TNF-α production was observed with the plasma samples from subjects with SBS (Figure 2A–C). SBS plasma was 2–3-fold more effective at suppressing TNF-α in the culture supernatant compared to plasma from HC. Furthermore, the TNF-α inhibition was similar across all the tested *E. coli* strains, with the highest inhibition observed with the SBS2 plasma sample (Figure S2B,C). For IL-6, HC plasma samples had no significant effect on IL-6 production induced by *E. coli* strains (Figure S2D–F), except for controls 3 and 4, which elicited a significant inhibition of IL-6 production against *E. coli* O86:B1 strain (Figure S2F). Irrespective of the *E. coli* strain, all the plasma samples from SBS subjects significantly (*p* < 0.05) inhibited the production of IL-6 when compared to splenocytes stimulated by *E. coli* alone (Figure S2D–F). Compared to HC samples, SBS plasma samples significantly inhibited IL-6 production only against the K1 strain (Figure 2E), while no significant difference was found for other *E. coli* strains (Figure 2D,F). Overall, our data suggest that plasma from the SBS subjects is more effective at neutralizing *E. coli*-induced pro-inflammatory cytokine responses in vitro.

### 2.2. Effect of Plasma from SBS and HC Subjects on Pro-Inflammatory Cytokine Levels in an E. coli Sepsis Murine Model

**Figure 3:**
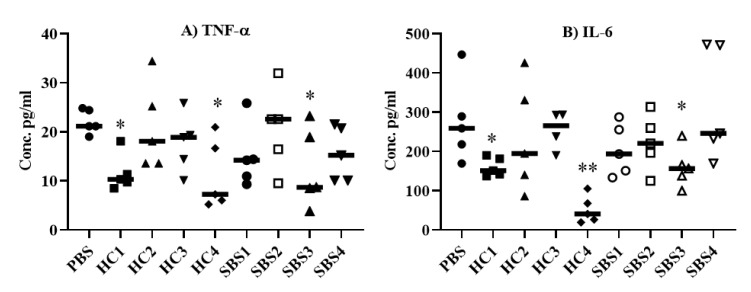
Anti-inflammatory effect of plasma from SBS and HC subjects on *E. coli* sepsis. Mice administered intravenously with PBS, SBS plasma or HC plasma were challenged 16 h later with an LD_100_ dose (5 × 10^7^ CFU) of *E. coli* ATCC 25922 strain intraperitoneally. Blood from each mouse was collected 22 h later and TNF-α and IL-6 levels in plasma were determined by ELISA. Data are presented as median with each data point representing one mouse. Non-parametric Mann-Whitney T-test was applied to analyze the statistical significance. * *p* < 0.05, ** *p* <0.001. SBS; short bowel syndrome, HC; healthy controls.

To assess the effect of SBS plasma on sepsis-mediated proinflammatory cytokine responses, we treated mice intravenously with PBS, plasma from HC, or plasma from SBS subjects; then, the mice were injected with an LD_100_ dose *E. coli* (5 × 10^7^ CFU) administered intraperitoneally. The mice that received PBS alone showed a robust induction of TNF-α (Figure 3A) and IL-6 (Figure 3B) production, while the treatment of mice with HC1, HC4, SBS1 or SBS3 led to substantial inhibition of these cytokines (Figure 3A,B). Although no significant difference was observed between SBS4 and PBS treatments, there was a trend towards inhibition of TNF-alpha, although this was not found in the IL-6 in SBS4 treatment group.

We also evaluated the effect of SBS and HC plasma samples in a model of lower acuity *E. coli* sepsis. Mice (*n* = 3–4) pretreated with either SBS or HC plasma samples were challenged with a sub-lethal dose of *E. coli* (1.5 × 10^7^ CFU), then sacrificed at 24 h for analysis of IL-6, TNF-α and IL-1 β in the mouse plasma and spleen (Figure S3). In plasma, we could detect only IL-6, which was statistically non-significant among treatment groups (Figure S3A). In contrast, all three measured cytokines were detected in spleen homogenates. The levels of TNF-α (Figure S3B) and IL-6 (Figure S3D) were non-significant among treatment groups, except for the HC4 treatment group, which showed significantly (*p* < 0.05) lower IL-6 levels compared to the mice treated with PBS alone. Although the levels of IL-1β did not reach statistical significance among treatment groups, mice treated with HC1, HC4, SBS1, SBS2, and SBS3 showed a trend of inhibition (*p* = 0.057) compared to the mice that received PBS alone (Figure S3D). We also looked at CFUs in spleens 24 h post-challenge (Figure S3G). Although CFUs were statistically non-significant among treatment groups, there was also a trend toward lower CFUs in mice treated with either HC or SBS plasma samples. Overall, our data indicate that SBS and HC plasma have potential anti-inflammatory properties in vivo.

### 2.3. Effect of Plasma from SBS and HC Subjects on Mortality Induced by E. coli in a Murine Sepsis Model

**Figure 4:**
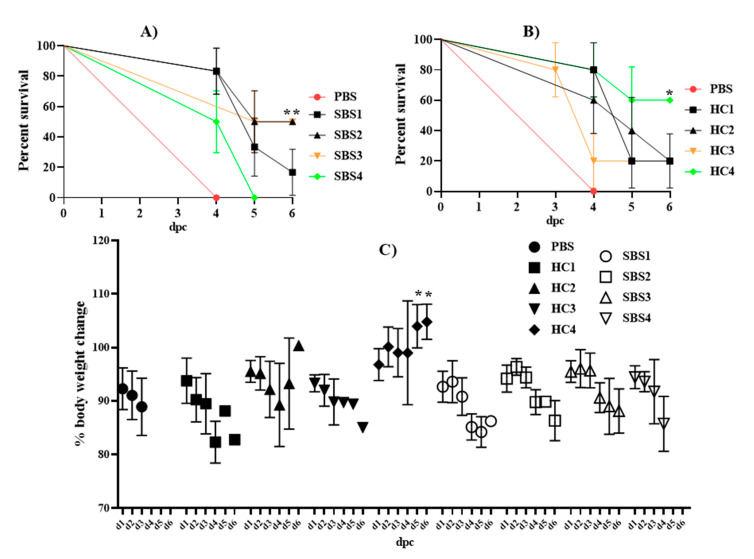
Effect of plasma from SBS and HC subjects on *E. coli* sepsis-induced mortality. Mice were treated intravenously with PBS, SBS plasma or HC plasma and, 16 h later, intraperitoneally challenged with LD_100_ *E. coli* ATCC 25922 strain. Post-challenge, mortality over 6 days was recorded, and survival distribution among the groups was determined using a log-rank Mantel–Cox test (**A**,**B**). Body weight was monitored daily and presented as mean ± SD (**C**). dpc: days post-challenge. * *p* < 0.05. ** *p* < 0.01. SBS: short bowel syndrome, HC: healthy controls.

To investigate whether plasma from SBS subjects can prevent sepsis-induced mortality, we treated mice with PBS, HC plasma, or SBS plasma followed by infection with a lethal dose of *E.* *coli* (5 × 10^7^ CFU). Treatment with PBS alone resulted in 100% mortality on day 4, while treatment with plasma from either SBS (Figure 4A) or HC (Figure 4B) provided a marked survival benefit against lethal sepsis. Mice that were treated with SBS2 or SBS3 plasma showed 50% (3 out of 6) survival, while SBS1- and SBS4-plasma-treated mice had only 16.6% (1 out of 6) and 0% (6 out of 6) protection, respectively. Excepting the HC4 treatment group, which led to a 60% (3 of 5) survival rate, treatment with all other HC plasma samples showed a 20% (1 out of 5) protection rate. We also measured the body weight of mice following the LD_100_ *E. coli* challenge. The results show that mice treated with SBS1, SBS2, or SBS3 initially resisted any changes in body weight until day 3, while all other treatment groups, except HC4, showed a substantial loss in body weight from day 1 post-challenge (Figure 4C). Treatment with HC4 plasma did not affect the body weight of mice following the LD*_100_ E. coli* challenge. Overall, our data indicate that plasma from both SBS and HC subjects can have a mitigating effect against sepsis-induced mortality.

## 3. Discussion

Despite the substantial progress made in the understanding of the pathophysiology of sepsis, there are no effective immunomodulatory therapies available to date for treatment of GN sepsis. Many previous efforts to control the overwhelming inflammation related to sepsis have been met with failure [33]. The present study used a well-established model of GN *E. coli* sepsis to investigate whether plasma from SBS patients could mediate protection against sepsis-induced mortality in mice. The relative lack of clinically severe symptoms exhibited by SBS patients with GN sepsis provided the rationale underlying our hypothesis. SBS plasma inhibited the secretion of proinflammatory cytokines induced by LPS or multiple clinical *E. coli* strains in vitro. Intravenous treatment with selected SBS and control plasma also inhibited sepsis-associated cytokines, and reduced mortality in mice challenged with a reproducibly lethal dose of *E. coli*.

Polyclonal intravenous immunoglobulins (IVIG), a pooled preparation of IgG antibodies, are frequently used in severely ill septic patients, due to their unique functions in eradicating pathogens and neutralizing toxins [34,35]. However, multiple IVIG clinical trials demonstrated inconsistent beneficial effects, possibly accounted for by the use of preparations from heterogeneous populations, lacking in specific protective immunity against sepsis-causing bacteria. More recent data have supported the additional benefits of using IVIG preparations specific to the causative agent related to sepsis [36,37]. Due to the frequent bacterial translocation with enteric GN pathogens, we hypothesized that SBS patients would have robust neutralizing antibodies to GN bacteria. Consistent with previous reports [27,38], we found a serum IgG antibody for LPS and *E. coli* strains in SBS as well as in healthy subjects (data not shown), suggesting that systemic B cell responses to gut-derived microbial antigens commonly occur irrespective of the state of the host. Plasma samples from SBS subjects have more profound inhibitory effects on LPS- and *E. coli*-mediated IL-6 and TNF-α production by host cells when compared to samples from HC subjects in vitro. We observed lower levels of pro-inflammatory cytokines in mice treated with select plasma samples from either SBS or HC subjects, which may explain the protection against sepsis-induced mortality. We demonstrated that mice receiving SBS plasma therapy survived longer than PBS-treated mice and that two of the four SBS plasma samples resulted in 50% protection against sepsis-induced mortality. However, the benefits against sepsis-induced mortality were also observed in mice treated with plasma samples from the HC subjects. Notably, among all the plasma-treated groups, the HC4 treatment group showed the lowest levels of cytokine inflammation and the most protection against sepsis-induced mortality, suggesting that some healthy subjects could elicit potent antibody responses against the gut microbes. This finding was in agreement with the previous report, which demonstrated that commensal-specific IgG antibodies mediate efficient protection against the translocated symbionts or the invasive enteric pathogens in systemic infection [38]. The variations in protection induced by different plasma samples are likely multi-factorial for various reasons, including age, microbiome diversity, comorbidities, and genetic polymorphisms; all of these could contribute to the development of B cell responses, unique to an individual. Therefore, some individuals might generate highly protective antibodies, which are more capable of performing multiple functions, including phagocytosis, activation of the complement system, efficient neutralization of endotoxins, and activation of various innate immune cells, which might explain why in vivo protection varied among treatment groups. Moreover, the heterogeneous presentations of sepsis, characterized by highly complex and individualized immune responses, suggest that there will not be a single “silver bullet” that can effectively treat sepsis. A study by Speer et al. showed that the addition of pentoxifylline, a phosphodiesterase inhibitor, to antibiotics in murine *E. coli* sepsis promoted anti-inflammatory milieu in plasma and organs without promoting bacterial growth [39], indicating that adjunctive therapies might be beneficial to manage sepsis-induced inflammation and mortality. The above study supports our proposal that plasma samples from SBS subjects with antibodies specific to sepsis-related microbes might be a safe and effective anti-inflammatory adjunctive therapy to manage sepsis-induced inflammation and mortality.

This study set out to test whether plasma from SBS subjects would provide robust protection against GN sepsis compared to plasma from healthy subjects. Unexpectedly, we did not see a relative lack of clinically significant symptoms in mice that received SBS plasma compared to mice that received healthy human plasma. It is possible that this lack of difference is due to the age of the plasma donors—older healthy plasma donors versus pediatric SBS plasma donors. Additionally, the results may have been affected by the heterogeneity within the SBS population, as subjects differed in the extent of PN dependence, number of known central line infections, time since last central line infection, isolated organisms, and time since last antibiotic exposure. This is further complicated by the inadequate methods of assessing intestinal health in subjects with SBS [40,41]. GN sepsis in mice was induced with a supraphysiologic inoculum of *E. coli* not seen in human disease and, therefore, may not accurately reflect the LPS-neutralizing antibody activities noted in the in vitro splenocyte assay. We favor the explanation that immune cells in SBS subjects, other than antibodies, conferred additional protection against overwhelming inflammation during GN sepsis. For example, limited human studies have shown that gut resection negatively impacts systemic immune responses, especially T cell responses [31,42]. Such studies have shown that patients with SBS have dysfunctional T cell responses, characterized by reduced cytokine and proliferative responses upon antigenic stimulation. Furthermore, regulatory T cells (Tregs) are found in higher proportions in SBS patients than in match-controlled subjects, indicating that SBS patients may have multiple immune evasion mechanisms to prevent a sepsis-like situation. Additional potential innate immune adaptation, for example, M1 to M2 conversion, has not been well studied. Understanding the fundamental biological processes governing the phenomenon of immune tolerance in SBS patients, including the interplay between the host and microbiome, would lead to a better understanding of the mechanisms underlying lack of SBS patient reactivity to GN sepsis, and could provide insight into the development of novel therapies against sepsis and septic shock, some of which may include either targeted therapeutic approaches by immunomodulation, alterations of the microbiome with probiotics, or a combination of both [43,44,45,46].

## 4. Conclusions

We show that intravenous treatment with plasma from SBS as well as healthy subjects can improve host survival in mice challenged with a lethal dose of *E. coli*, although without a significant difference in efficacy between SBS and healthy plasma. Future studies should evaluate plasma and PBMC samples from a large cohort of SBS patients, which could provide information on a more effective management of GN sepsis, beyond the use of antibiotics. To the extent that sepsis-induced organ damage and mortality is driven by proinflammatory cytokines, our study warrants an investigation into SBS plasma samples in combination with current standard sepsis therapies to determine if sepsis-induced inflammation and mortality could be improved.

## 5. Materials and Methods

### 5.1. Bacterial Strains and Culture Conditions

Clinical *E. coli* strains, including ATCC 25922, K1 RS218, and O86:B1, were used in this study. The *E. coli* strains were grown in 5 mL of Luria Bertani (LB) broth with agitation to a log phase (OD600nm = 0.65–0.7) at 37 °C. The bacterial culture was centrifuged at 2400× *g* for 5 min and the pellet was washed twice with sterile phosphate-buffered saline (PBS; pH = 7.40). The bacterial pellet was finally resuspended in PBS to the desired colony-forming units (CFU) for further use.

### 5.2. In Vitro Studies with Plasma from SBS Subjects

CD1 mouse splenocytes (*n* = 3) were used to study the effect of plasma from SBS and HC subjects on the cellular immune responses mediated by LPS and *E. coli* in vitro. Subjects were recruited at outpatient pediatric gastroenterology clinic visits. Inclusion criteria included children aged 5–18 years with anatomic SBS and central line access for chronic PN use. Exclusion criteria included presence of fever within 30 days prior or antibiotic administration within 7 days prior to the visit. The demographics and characteristics of the SBS subjects are provided in Table 1. Adults aged 18 years or older with no underlying acute or chronic illness were utilized as control subjects; again, those who had fever within 30 days or received antibiotics within 7 days prior to recruitment were excluded.

LPS (1 µg/mL) or *E. coli* (1 × 10^8^ CFU/mL) was incubated with 5%, 10%, or 20% of plasma from either HC or SBS subjects at room temperature, under continuous shaking for 30 min. The mixture containing 250 ng of LPS or 1 × 10^6^ CFU of *E. coli* was then applied to the splenocytes (1 × 10^6^ cells), followed by incubation of the cells at 37 °C under 5% CO2. Cells stimulated with either LPS alone or with the *E. coli* strain alone were used as positive controls and media-treated cells were used as the negative controls. The splenocytes were plated in a Falcon^®^ 96-well tissue culture plate (Catalog no. #353072, corning incorporated, USA) and cultured in complete RPMI medium (Gibco, ThermoFisher Scientific, Waltham, MA, USA) supplemented with 10% fetal bovine serum and 1x penicillin–streptomycin antibiotics solution (Catalog#P4333, Sigma-Aldrich, St. Louis, MO, USA). After 24 h incubation, the cells were centrifuged at 450× *g* and the culture supernatant was collected for analysis of the proinflammatory cytokines, including IL-6 and TNF-α, by a solid-phase sandwich enzyme-linked immunosorbent assay (ELISA; Biolegend, San Diego, CA, USA).

### 5.3. In Vivo Efficacy of Plasma from SBS Subjects on Sepsis-Induced Mortality

All animal studies were approved under the guidelines of the University of California San Diego (UCSD) Institutional Animal Care and Use Committee. Outbred 6–8-week-old female CD1 mice (The Charles River Laboratory) were housed in an animal facility at UCSD with a standard of care as per federal, state, local, and NIH guidelines.

Before SBS plasma samples were tested, the lethal dose (LD) was determined in groups of 4 mice inoculated with different CFU of *E. coli* ATCC 25922 strains, suspended intraperitoneally in 200 μL of 1×PBS (Figure S4). The inoculum that causes 100% mortality was defined as LD_100_ and used to study the efficacy of plasma samples on the survival of mice, following *E. coli* challenge. To evaluate the effect of plasma samples from SBS and HC on cytokine inflammation, mice were divided into 8 groups (*n* = 6) and administered retro-orbitally with 100 μL of plasma from either SBS or healthy subjects (*n* = 4). Mice that received PBS alone (*n* = 5) served as a positive control for sepsis-induced mortality. The retroorbital injection was performed under Isoflurane (Fluriso, Vet One) anesthesia. Sixteen hours after treatment with plasma samples, mice were challenged with LD_100_ *E. coli* intraperitoneally and bled 22 h later for quantification of proinflammatory cytokines, including TNF-α and IL-6, in mouse plasma obtained from the heparinized blood, spun at 10000 rpm for 10 min. To evaluate the effect on survival, the mice were pretreated as previously discussed, and then infected and monitored for survival over 6 days.

To further evaluate the effect of HC and SBS plasma samples on the induction of proinflammatory cytokine responses in a less severe sepsis model, mice were treated retro-orbitally with 100 µL of either HC or SBS plasma, followed by challenge with an intraperitoneal sub-lethal dose of *E. coli* (1.5 × 10^7^ CFU). Twenty-four hours later, plasma and spleens were collected for cytokine estimation and CFU enumeration. For CFU enumeration, spleens were homogenized in 200 µL of sterile PBS in microcentrifuge tubes, and then serially diluted with PBS (from 10^−1^ to10^−4^) and plated onto agar plates. After 24 h of culture, bacterial colonies were counted.

### 5.4. Cytokine ELISA

IL-6, IL-1β and TNF-α cytokine levels were measured by a solid-phase sandwich ELISA using commercially available cytokine ELISA kits (Biolegend, San Diego, CA, USA). The assay was performed in duplicate or triplicate, as per manufacturer’s instructions. The culture supernatants, blood plasma samples, or splenic homogenates were diluted 1:1 with coating buffer (1%BSA + 1xPBS-Tween20) and used in the assay, along with the known concentration of cytokine standards (provided with the kits) in each ELISA plate. The plates were developed and read at optical density (OD) of 450 nm in a multimode microplate reader (PerkinElmer, Waltham, MA, USA). The standard curve generated from the OD of cytokine standards was used to determine cytokine levels in the samples.

### 5.5. Statistical Analysis

GraphPad prism version 8 was used to analyze the data. Cytokine data are presented as a median, and a non-parametric Mann–Whitney test was used to analyze the significant difference between treatment groups. Differences in survival (%) between treatment groups were compared by the use of a log-rank (Mantel–Cox) Chi square test. *p* values of <0.05 were considered significant.

## Figures and Tables

**Table 1:** Demographics and characteristics of subjects with SG.

Characteristics	Subject 1	Subject 2	Subject 3	Subject 4
Age (years)	5	9	14	9
Sex	F	M	F	F
Diagnosis	Malrotation, Midgut volvulus, Jejunal atresia	Necrotizing enterocolitis, Jejunal perforations	Large and small bowel atresia, Persistent omphalovitelline duct	Gastroschisis
Remaining Small Bowel Length	Unknown. Noted to have resection of 3 cm jejunum, 6 cm ileum	63.6 cm	41 cm	45 cm
PN ^a^ dependence	9%	14%	72%	100%
Current Central Line (Duration)	Broviac (59 months)	Port-a-Cath (12 months)	Broviac (13 months)	Broviac (39 months)
Number of Line Infections ^b^	0	2	1	21
Time Since Last CLABSI, past organisms Isolated	NA	8 years Lactobacillus	7 years corynebacterium spp	1 month *E. coli, K.oxytoca, S.epidermidis, S.haemolyticus, S. hominis, S.gallolyticus, E. faecalis*
Antibiotic Therapy for SIBO ^c^ or Prokinetic Effects	Yes	Yes	Yes	No
Days from Last Antibiotic Exposure	7	14	60	35

^a^ Parenteral Nutrition. ^b^ Confirmed within our institution. Subjects 3 and 4 received prior care at outside facilities. Subject 3 was reported to have multiple line infections of a previously unknown number. ^c^ Small Intestinal Bacterial Overgrowth.

## Data Availability

HYPERLINK “C:\\Users\\nramchandar\\Downloads\\Deidentified” Deidentified data is available from the authors upon reasonable request.

## Supplementary Materials

The following are available online at https://www.mdpi.com/article/10.3390/pathogens10081021/s1, Figure S1: LPS neutralization in vitro. *E. coli* LPS incubated with various concentrations of plasma from subjects with SBS or HC, including 5% (A&B) and 10% (C&D), was applied to mouse splenocytes cultured in RPMI media at 37 °C. TNF-α and IL-6 from the culture supernatants were quantified by a solid-phase sandwich ELISA performed in duplicate or triplicate wells. Data are presented as mean ± SD, and a parametric unpaired T-test was applied to analyze the statistical significance. * *p* < 0.05. SBS: short bowel syndrome, HC: healthy controls. Figure S2: Anti-inflammatory effect of plasma from SBS or HC subjects in *E. coli*-stimulated splenocyte cultures. *E. coli* strains ATCC 25922 (A&D), K1 RS218 (B&E), and O86:B1 (C&F) were incubated with either plasma from SBS or HC and applied to the mouse splenocytes cultured at 37 °C. TNF-α and IL-6 pro-inflammatory cytokines were quantified in the culture supernatant by a solid-phase sandwich ELISA performed either in duplicate or triplicate wells. Data are presented as mean ± SD, and a parametric unpaired T-test was applied to analyze the statistical significance. **p* < 0.05. SBS: short bowel syndrome, HC: healthy controls. Figure S3: Anti-inflammatory effect of plasma from SBS and HC subjects on sub-lethal *E. coli* sepsis. Mice (*n* = 3–4) administered intravenously with PBS, SBS plasma or HC plasma were intraperitoneally challenged 16 h later with 1.5 × 10^7^ CFU of *E. coli* ATCC 25922 strain. Blood from each mouse was collected 24 h later, and TNF-α, IL-1β, and IL-6 levels in plasma and spleen were determined by ELISA. Data are presented as median, with each datapoint representing one mouse. Non-parametric Mann–Whitney T-test was applied to analyze the statistical significance. **p* < 0.05, **< 0.001. SBS: short bowel syndrome, HC: healthy controls. Figure S4: LD100 evaluation for E. coli ATCC 25922 strain in a peritonitis-induced sepsis model. Groups of 4 mice were injected with different CFUs of an *E. coli* strain 25922, suspended intraperitoneally in 200 µL of 1xPBS. The mice were monitored over seven days, and the number of deaths in each group was recorded daily. The survival data were evaluated using a log-rank Mantel–Cox test. dpi; days post-infection. *p* < 0.001.
