# Supplementary figures and images for: Anti-Inflammatory Properties of Plasma from Children with Short Bowel Syndrome
Source: Pathogens. 2021 Aug 13;10(8):1021. doi: 10.3390/pathogens10081021 (PMC8400962; doi:10.3390/pathogens10081021)

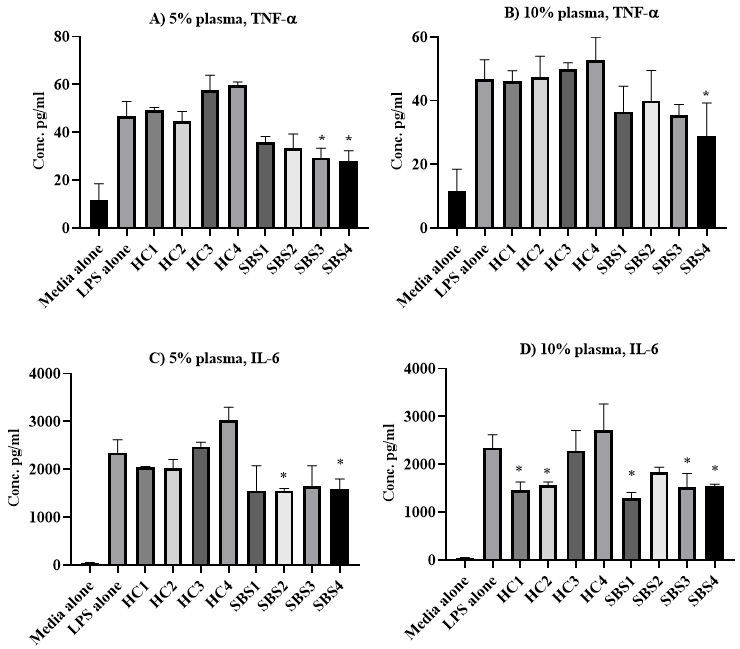

Supplement: Supplementary file 1 [file pathogens-10-01021-s001.zip › pathogens-1328520-supplementary/supplementary/Supplementary Figure S1.tif]

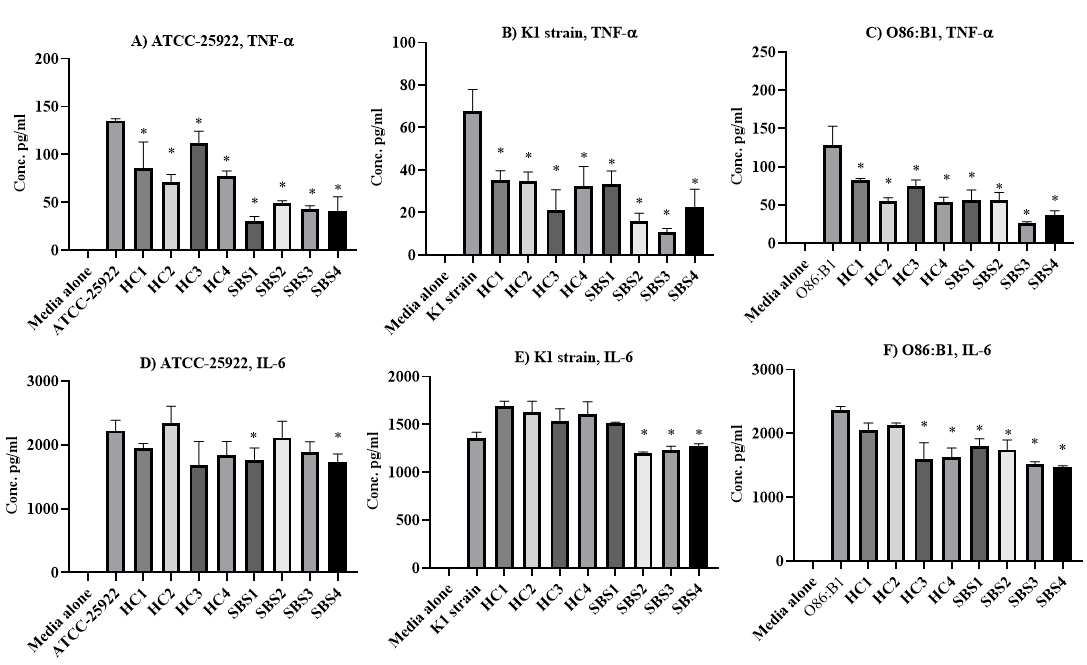

Supplement: Supplementary file 1 [file pathogens-10-01021-s001.zip › pathogens-1328520-supplementary/supplementary/Supplementary Figure S2.tif]

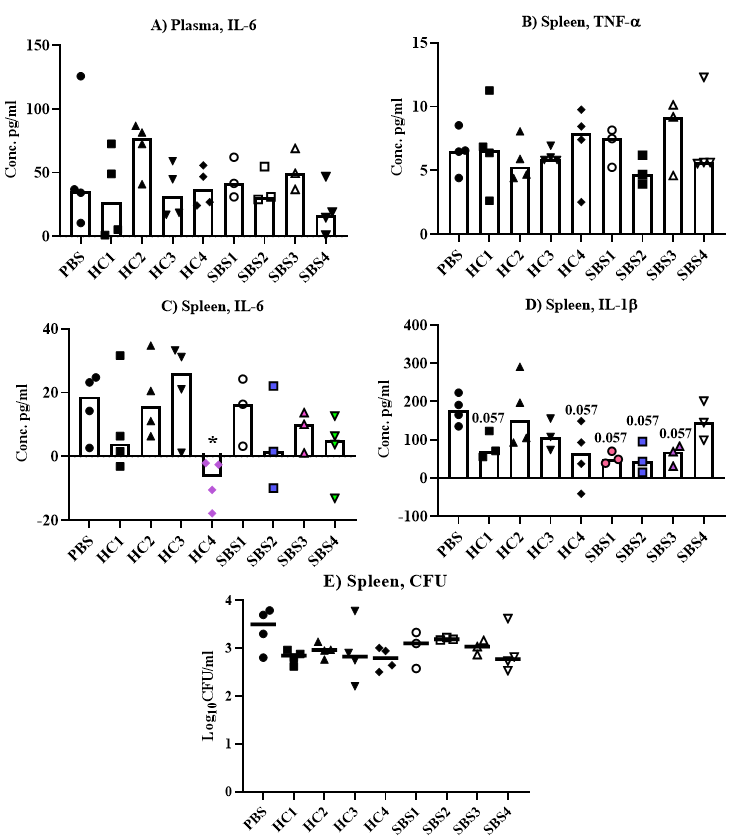

Supplement: Supplementary file 1 [file pathogens-10-01021-s001.zip › pathogens-1328520-supplementary/supplementary/Supplementary Figure S3.tif]

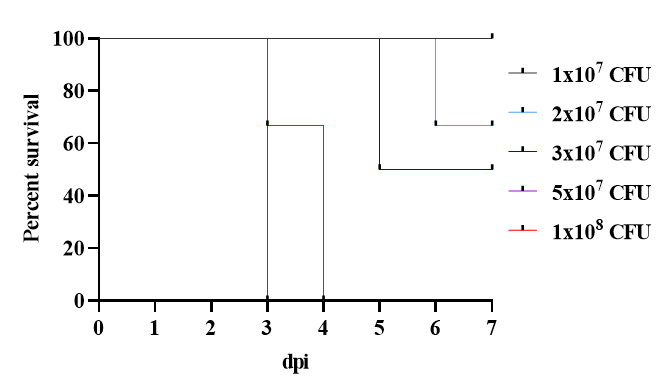

Supplement: Supplementary file 1 [file pathogens-10-01021-s001.zip › pathogens-1328520-supplementary/supplementary/Supplementary Figure S4.tif]
